# If not now, when?

**DOI:** 10.1192/bjb.2020.37

**Published:** 2021-02

**Authors:** Norman A. Poole

**Affiliations:** Department of Neuropsychiatry, St George's Hospital, South West London and St George's Mental Health NHS Trust, UK

**Keywords:** COVID-19, mental health services, resilience

## Abstract

The editor of the *BJPsych Bulletin* reflects on the extraordinary recent events triggered by the COVID-19 pandemic. Mental health professionals are at the front line of managing the pandemic and emergency changes should lead to a much needed refocus on what is really vital. In these unsettling times we ought to review how we manage the crisis, and its aftermath, both personally and professionally.

## 21 March 2020

My 3-year-old daughter woke this morning with a cough. Rather sweetly, she claimed she'd ‘Caught hold of the cough’ which she knows is making people ill. Instead of the group cycle I'd planned, I rode out on my bike alone, giving others an acceptably wide berth. Well, I say acceptably wide, but how wide is that? Two metres or more? Should I even have been out exercising? Is it a cold or COVID? How worried should I be? Pedalling into a cold northerly squall, it suddenly dawned on me: ‘I'm scared’. Not so much for myself – although perhaps I'm not yet willing to admit that – but for my daughter, my family, friends, their families, colleagues and, of course, our patients. We are taught that insight in psychosis is impaired, but I've often found anxiety to be less well recognised by patients, and me it turns out, than the textbooks tell us.^1^ It was an unsettling discovery because at that moment I also realised how powerless I am.

The neuropsychiatry team at St George's where I work had spent the previous week switching to remote clinics, mainly from home, but also seeing neurology in-patients at St George's Hospital. We learned that the liaison psychiatry service, led by the unflappable Marcus Hughes, had split into red and green teams; the former working exclusively in the new COVID-19 unit. ‘How noble?’, I thought. ‘How long will they last?’, I fretted. Everyone accepts that at some point they will have to take their turn in the red team. We heard how our in-patient colleagues on the mental health wards are also dividing themselves into teams and containing units to mitigate the virus's spread. ‘How ironic?’, I mused. Doctors were first sent to the asylums some 200 years ago to prevent contagion seeping out into the community at large.^2^ Now we are struggling to do the opposite. Community teams are restructuring services on the hoof to maintain care despite all the limitations imposed on them. As I write, it still feels like a phoney war, yet so unnerving to watch our futures unfolding before us in daily despatches from the frontlines in Italy and Spain. In this war against an invisible enemy the frontline is long, and thin. And mental health professionals are as much part of it as anyone.

I personally hate Churchill's quip about ‘Not letting a good crisis go to waste’, which so blithely ignores human cost and personal tragedies. Yet I am surely not alone in believing that COVID-19 must change how we deliver mental healthcare for good. The pandemic, from which we will hopefully recover, and our catastrophic mismanagement of the environment are not unrelated events. Pathogens are increasingly likely to cross species barriers as we pillage natural habitats.^3^ Tomlin's editorial^4^ draws attention to healthcare's contribution to poisonous greenhouse emissions and a previous article in this journal described the damage that excreted SSRI medications could be wreaking on our marine environment.^5^ We are currently working towards a special edition of the *BJPsych Bulletin* on the climate crisis and psychiatry, which will highlight the problems and point to some solutions. In this vein, I am hopeful that many of the new ways we are working – telepsychiatry^6^ and stripped-back bureaucracy – will outlive the current crisis. Psychiatry must resist successive governments’ fantasy that individual risk can be managed on the basis of population-level statistics.^7,8^ It can't, and we must say so. The reality is, we would probably have the workforce to deliver a world-class mental health service if everyone wasn't so tied up inputting pointless data. If not now, when?

The virus has also exposed glaring injustices in our society. Some have the resources to weather the storm either through accumulated wealth or the luxury of being able to work from home. Many others live hand-to-mouth in insecure jobs while paying extraordinary housing costs. How will they fare? The sacrifices being made across the board must lead to a rewriting of the social contract, as happened after the Second World War. The debts currently being accrued cannot be repaid with regressive income taxes while personal and corporate wealth remains undertaxed.^9^ Unlike post-2008,^10^ corporate bailouts must come with conditions that benefit the majority. Later this year, with Peter Byrne's support, *BJPsych Bulletin* will publish a themed edition on inequality as a major source of mental disorder. I'd say it is timely, but we have known this stuff for years yet not always, it seems, accepted the corollary: psychiatry must argue unflinchingly for a fairer society. If not now, when?

This crisis will be demanding on us all, but social distancing also provides opportunities. As I cycled alone, which I expect to be doing a lot more of, I realised just how addicted I'd become to the relentless news cycle. I've resolved to limit my intake to once a day. Much better to repurpose these new uninvited evenings spent at home. Sadly, I doubt it'll be to learn the piano but in these strange times I'm opening up to music recommended by others (please send some suggestions!) and have been live streaming gigs from my favourite venue (Café Oto, if you're interested: https://www.cafeoto.co.uk). There are various writing projects that I aim to complete before unwinding with movie nights. I have already had an evening in a virtual pub and reconnected with long-lost friends. All the while knowing that these are mere displacement activities to manage a gnawing fear. It will be harder still for the completely self-isolating over-70s. Many I know are self-organising to support the vulnerable locally, and it will be a testament to our society if these activities endure. Closer to home, I've started a book club with my mum to help keep her spirits up in the long months ahead and allow us to chat about something, anything, else. And there are plenty of books that have sat on my to-do list far too long. My apocalyptic reading starts here: *If Not Now, When?*^11^

## About the author

**Norman A. Poole** is Editor of the *BJPsych Bulletin* and a consultant neuropsychiatrist at St George's Hospital, South West London and St George's Mental Health NHS Trust, UK.
